# Optimum Extraction Condition of *Clitorea ternatea* Flower on Antioxidant Activities, Total Phenolic, Total Flavonoid and Total Anthocyanin Contents

**DOI:** 10.21315/tlsr2020.31.2.1

**Published:** 2020-08-06

**Authors:** Nurjamalina Fasihah Jaafar, Muhammad Ezzudin Ramli, Rabeta Mohd Salleh

**Affiliations:** Food Technology Division, School of Industrial Technology, Universiti Sains Malaysia, 11800 USM Pulau Pinang, Malaysia

**Keywords:** Antioxidant Activity, Flavonoid, Anthocyanin, *Clitoria ternatea*, Response Surface Methodology, Aktiviti Antioksidan, Flavonoid, Antocianin, *Clitoria ternatea*, Metodologi Respons Permukaan

## Abstract

*Clitoria ternatea* is a herbaceous plant with many health benefits. Extraction is crucial to obtain its bioactive components which contribute to its antioxidant properties. Therefore, this study was conducted to find an optimum extraction condition of *C. ternatea* flower on total phenolic content (TPC) and antioxidant activity (2,2-diphenyl-1-picrylhydrazyl (DPPH) free radical-scavenging activity) as well as to determine its total flavonoid content (TFC) and anthocyanin content based on the optimum extraction condition generated by Response Surface Methodology (RSM)-Design Expert 7.1.5. TPC, TFC and total anthocyanin of *C. ternatea* were conducted by Folin Ciocalteu (FC), calorimetric assay and pH differential method, respectively. The ranges of selected independent variables were ethanol concentration (30°C–90% v/v), time (60–120 min) and temperature (30°C–70°C). The optimum extraction condition was obtained at 39.62% v/v ethanol concentration, 90 min and 44.24°C. However, these values were slightly adjusted according to the convenience of equipment to operate in which ethanol concentration was adjusted to 37% v/v, time remain at 90 min and temperature at 45°C. The predicted values of TPC and DPPH radical scavenging activity were 41.60 mg GAE/g dry samples and 68.12% inhibition and were experimentally verified to be 41.17 ± 0.5 mg GAE/g dry samples and 63.53 ± 0.95% inhibition of TPC and DPPH radical scavenging activity respectively. This result has showed RSM can optimise TPC and radical scavenging activity of *C. ternatea*. Upon the optimum condition, the TFC determined was 187.05 ± 3.18 mg quercetin/g dried sample which was higher than TPC and the total anthocyanin content was 28.60 ± 0.04 mg/L. Hence, the extractable phenolic, flavonoid and anthocyanin compounds indicated that *C. ternatea* is a good source of natural antioxidant.

## Abstract (ms)

*Clitoria ternatea* adalah tumbuhan herba yang banyak kebaikan kesihatan. Pengekstrakan adalah penting untuk mendapatkan komponen bioaktif yang menyumbang kepada sifat antioksidannya. Oleh itu, kajian ini dijalankan untuk mendapatkan keadaan pengekstrakan yang optimum ke atas bunga *C. ternatea* terhadap jumlah kandungan fenolik (TPC) dan aktiviti antioksidan (2,2-difenil-1-picrylhydrazyl (DPPH) aktiviti pemerangkapan radikal bebas) serta menentukan jumlah kandungan flavonoid (TFC) dan antosianin berdasarkan keadaan pengekstrakan optimum yang dihasilkan oleh Metodologi Respons Permukaan (RSM)-Design Expert 7.1.5. TPC, TFC dan jumlah antosianin *C. ternatea* masing-masing ditentukan oleh Folin Ciocalteu (FC), ujian kalorimetri dan kaedah pembezaan pH. Pembolehubah bebas yang dipilih adalah kepekatan ethanol (30%–90% v/v), masa (60–120 min) dan suhu (30°C–70°C). Kondisi pengekstrakan optimum diperolehi pada kepekatan ethanol 39.62% v/v, 90 min dan 44.24°C. Walau bagaimanapun, nilai-nilai ini telah diselaraskan mengikut kemudahan peralatan untuk beroperasi iaitu ethanol diselaraskan kepada 37% v/v, masa kekal pada 90 min dan suhu pada 45°C. Nilai ramalan kandungan fenolik dan aktiviti pemerangkapan radikal DPPH adalah 41.60 mg GAE/g sampel kering dan 68.12% perencatan radikal dan experimen pengesahan keadaan optimum mendapati keputusan TPC ialah 41.17 ± 0.5 mg GAE/g sampel kering dan keputusan pemerangkapan radikal DPPH ialah 63.53 ± 0.95%. Keputusan ini menunjukkan bahawa RSM dapat mengoptimumkan pengekstrakan TPC dan aktiviti antioksidan *C. ternatea*. Berdasarkan keadaan pengektrakan yang optimum, jumlah kandungan flavonoid yang ditentukan ialah 187.05 ± 3.18 mg quercetin/g sampel kering yang iaitu lebih tinggi daripada kandungan TPC dan jumlah kandungan antocianin adalah 28.60 ± 0.04 mg/L. Oleh itu, komponen fenolik, flavonoid dan antosianin yang boleh diekstrak menunjukkan bahawa *C*. adalah sumber antioksidan semula jadi yang baik.

**Highlights**

- Several crucial factors, such as the solvent, extraction time and temperature should be studied to determine the optimum extraction condition and high amount of bioactive compounds and antioxidants in *Clitoria ternatea* flower.
- The optimum condition generated by central composite design (CCD) of Response Surface Methodology (RSM) was 36.98% of ethanol concentration, 90 min of extraction time and 44.27°C of extraction temperature.
- The extractable phenolic, flavonoid and anthocyanin compounds indicated that *Clitoria ternatea* is a good source of natural antioxidant.

## INTRODUCTION

Recently, various types of medicinal or supplements are being consumed by people who are largely concerned about their health. The antioxidant contents of these supplements are commonly determined because of their beneficial effects. Antioxidant can prevent the risk of getting cancer by fighting or scavenging free radicals (Spigno *et al*. 2007). Several food manufacturers may also use synthetic antioxidant to prolong their products’ shelf life (Sies *et al*. 1992). The active compounds in plants have been widely researched due to their effects on health, especially on the phenolic content and antioxidant activity.

Different extraction methods are commonly selected for different purposes and suitability. For instance, modern extraction is usually used to increase the yield of the extracted compound at low cost (Bucić-Kojić *et al*. 2011). Nevertheless, the antioxidant extraction is not only dependent on the extraction method but also on the solvent used because antioxidants exist in different chemical compositions, characteristics and polarities, which solubilise differently in different solvents (Turkmen *et al*. 2006).

The most common solvents being used in recovering antioxidants from plants is ethanol, methanol, acetone and ethyl acetate (Wang *et al*. 2008). Nevertheless, the effectiveness of solvent in extracting bioactive compounds from plants is different among different kinds of species. Approximately 100% ethanol extract of *Limnophila aromatica* results in the highest 2,2-diphenyl-1-picrylhydrazyl (DPPH) radical scavenging activity and the TPC of about 40.5 mg gallic acid equivalent/g defatted sample (Do *et al*. 2014). Furthermore, 30% ethanol and acetone extracts are the most efficient condition in extracting polyphenols in dried sage leaves (Dent *et al*. 2013). Solvents can possibly exert distinctive effects because different types of plants may exhibit different structures and polarities of the extracted bioactive components (Pandey & Tripathi 2013).

Response Surface Methodology (RSM) is mathematical and statistical analysis software that is used according to the fit of a polynomial equation of the experimental data to optimise the functional relationships between several variables and responses of interest (Khuri & Mukhopadhyay 2010). RSM is advantageous for optimisation when studying the effective interactions among multi-variables on the responses and reducing the required number of experiments (Bezerra *et al*. 2008).

Phenolic compounds are crucial for the antioxidant properties of plant extracts; these compounds extend the shelf life of food products incorporated with plant extracts (Borrás-Linares *et al*. 2015). Phenolics are phytochemicals that can be found in many kinds of colours fruits and vegetables. These compounds are also natural sources of antioxidants which help prevent cancer and fight against free radicals (Williams *et al*. 2004). Under the phenolic compound group, several types of phenolics include phenolic acids, flavonoids, stilbenes, coumarins and tannins.

Flavonoids also known as vitamin P (Crozier *et al*. 2006) are secondary plant metabolites that are directly involved in plant growth and development (Prasad *et al*. 2009). These metabolites, flavonoids can also be ingested by human and they commonly play vital roles as anti-inflammatory, anti-cancer and anti-allergic agents (Crozier *et al*. 2006). Flavonoids are bioflavonoids, which are natural polyphenol antioxidants in various plants (Holiman *et al*. 1996). These compounds contribute to plant colour, protecting plants from insects and microbes, and provide benefits to human health (Bravo 2009). Flavonoids can also be nutritionally beneficial by triggering enzymes that reduce certain risk of diseases such as cancer and age-related degenerative diseases (Carlo *et al*. 1999).

Anthocyanin is a flavonoid subclass that is water-soluble and stable in mild acids solution (Li *et al*. 2013). The anthocyanin colour is strongly dependent on its surrounding pH (Kungsuwan *et al*. 2014). Anthocyanin mostly exists as glycoside, which consists of aglycone cores (Kungsuwan *et al*. 2014). In butterfly pea petals, the identified aglycone core is kaempferol (Kazuma *et al*. 2003) which is present as positively charged oxonium ion in acidic solution or so called as flavylium catio (Kungsuwan *et al*. 2014). In low pH solution, the oxonium ion structure causes the conjugation of double bonds through three rings of aglycone moiety (Chen & Gu 2013).

*C. ternatea* is a flower commonly used by Malay people for herbs and cooking purposes (Kaisoon *et al*. 2011). Specifically, according to the International Legume Database and Information Service (1994), *C. ternatea* or also known as butterfly pea from the Fabaceae family exhibits medicinal properties. This flower’s petal is usually used as healthy beverage and food colourant worldwide (Gomez & Kalamani 2003). The petal is also widely used in making *nasi kerabu* and rice cakes as food colourant and replacement of synthetic colourant (McCann *et al*. 2007). These beneficial uses are attributed to that the amount of anthocyanin in blue pea butterfly higher than those in roselle and dragon fruit (Suppadit 2011). The anthocyanin in *C. ternatea* plant exists as delphinidin (Tanaka *et al*. 2009). This plant also contains high number of phytochemicals, which make it suitable for application in nutraceutical field. This plant is most ideally grown in soil with pH range of 5.5–8.9 (Morris 2008). Given its many benefits and application in Ayurvedic remedies, further study on *C. ternatea* should be carried out to determine the most suitable extraction condition and consequently obtain the highest amount of extracted bioactive compounds for health application. This study aims to determine the optimum extraction condition for *C. ternatea* flower and ascertain their TPC and antioxidant activity.

## MATERIALS AND METHODS

### Sample Collection and Preparation

Fresh samples of *C. ternatea* were collected from Lunas, Kedah. The voucher specimen number of *C. ternatea* herbarium USM is 11462. The flower was identified by Mr. Adnan Jaafar. The petals were separated from the sepal after being picked from its tree and then freezed overnight in refrigerator before being transferred into freeze dryer (Labconco, New York) at temperature of −50°C for three days to remove moisture and obtained dry powder for extraction. After the samples had been completely dried, samples where grounded into powder (Panasonic MX-898M-LW) and kept into vacuum package in desiccator for further analysis.

### Sample Extraction

Sample extraction method was performed as described by Dent *et al*. (2013) with slight modifications. About 0.1 g of freeze-dried samples was weighed into media bottles wrapped with aluminium foil, and then 10 mL of ethanol with concentrations of 30%, 60% and 90% was added into the bottles separately and was extracted for 60, 90 and 120 min at temperatures of 30°C, 50°C and 70°C on a horizontal water bath shaker (Memmert WB14, SV1422, Schwabach, Germany). The extracts were filtered through Smith No.101 filter paper and freshly analysed.

### Experimental Design

RSM-Design Expert 7.1.5 with three-level factorial central composite design (CCD) was used to determine the optimum extraction condition of *C. ternatea*. Three uncoded independent variables were identified, ethanol concentration (X1: 30%–90%), extraction time (X2: 60–120 minutes) and extraction temperature (X3: 30°C–70°C) on TPC (Y1) and 2,2-diphenyl-1-picrylhydrazyl (DPPH) free radical-scavenging assay (Y2). To identify the optimum extraction condition, both graphical and numerical optimisation were performed. Complete design with rotatable alpha lead to 20 runs of experiments. Six replicate runs at the centre points of the design were generated to estimate the pure error. All experiments were randomly performed to minimise the effect of unexplained variability in the observed responses due to systematic errors.

### Verification of Model

The optimum condition for the extraction of TPCs and antioxidant activity (DPPH) of *C. ternatea* depended on the factors being analysed, i.e. ethanol concentration, time and temperature which were determined by the second-order polynomial model of RSM. A series of solutions was generated, and verification was conducted according to desirability. The experimental values were to be compared with the predicted values of TPC and DPPH scavenging activity to predispose the validity of model.

## TPC

The TPC was determined by Nickavar *et al*. (2008) with slight modification. Approximately 0.1 mL fresh extracts were mixed with 2.5 mL Folin-Ciocalteau reagent (diluted 1/10) and incubated at room temperature for 10 min. About 2 mL of 7.5% w/v sodium carbonate was added into the mixture and allowed to stand for 30 min before the absorbance was read at 765 nm using UV-visible spectrophotometer (UV-160A, SHIMADZU, Kyoto, Japan). A calibration curve was prepared by using standard solution of gallic acid 0 to 100 mg L^−1^ concentration. Result was expressed as mg gallic acid/g dry sample.

### 2,2-diphenyl-1-picrylhydrazyl (DPPH) Free Radical-Scavenging Assay

The antioxidant activity of extracts was determined by using 2, DPPH free radical-scavenging assay according to Braca *et al*. (2002). The 0.1 mL extracts were added into 2.9 mL of 1 mM DPPH solution in the test tube wrapped with aluminium foil. The mixture of solution was incubated in darkness at room temperature for 30 min before the absorbance was read at 517 nm by using UV-vis spectrophotometer (UV-160A, SHIMADZU, Kyoto, Japan). The percent of inhibition was calculated by using the following formula:

% Inhibition=[(A control-A sample)/A control]×100%

## TFC

This analysis was carried out according to method described by Abu Bakar *et al*. (2009) with slight modification. About 0.5 mL of freshly prepared extract was mixed with 2.25 mL distilled water in a test tube wrapped with aluminium foil. Then, 0.15 mL of 5% w/v sodium nitrate solution was added into the mixture. The solution could stand for 6 min before 0.3 mL of 10% w/v aluminium chloride was added. After 5 min, 1.0 mL of 1 M sodium hydroxide was added, and the mixture was immediately vortexed. The absorbance was read at a wavelength of 510 nm by using UV-Visible spectrophotometer (UV-160A, SHIMADZU, Kyoto, Japan). A calibration curve by using standard solution quercetin was prepared and results were expressed as mg quercetin/g dry weight.

### Total Anthocyanin

Total monomeric anthocyanin was determined by using pH differential method according to Association of Official Analytical Chemists (AOAC) Official Method 2005.02 (AOAC 2000). The freshly prepared extract was properly diluted with pH 1.0 potassium chloride buffer and pH 4.5 sodium acetate buffers respectively in two different test tubes. The absorbance of test portion diluted with both pH 1.0 and pH 4.5 was determined at wavelength of 520 nm and 700 nm with the cell blank filled with distilled water. The absorbance value was determined by using the equation:

Absorbance (A)=[(A520nm-A700nm) pH 1.0-(A520 nm-A700 nm) pH 4.5]

The total anthocyanin was expressed as cyanidin-3-glucoside equivalent as shown in the equation:

$$\text{Total anthocyanin}\frac{\left(\text{mg}\right)}{\text{L}}=\frac{\text{A}\times \text{MW}\times \text{DF}\times 1000}{ɛ\times l}$$

Where;

MW = Molecular weight (448.8 g/mol for cyanidin-3-glucoside)
DF = Degree of freedom
ɛ = Molar extinction coefficient (26, 900 L/mol/cm for cyanidin-3-glucoside)
*l* = Path length (1 cm)

### Statistical Analysis

Analysis of variance (ANOVA) was conducted on both responses in RSM to analyse the significance of quadratic model and understand the mutual interactions between independent variables tested. The adjusted *R*^2^ is the corrected *R*^2^ after the elimination of unnecessary model term. The coefficient of variation (C.V) is the standard deviation in the form of percentage. Finally, to test the validation of optimum extraction condition generated by RSM, the significance level of both predicted and experimental values of TPC and DPPH were tested by using one sample t-test based on IBM SPSS statistic 23.

## RESULTS AND DISCUSSION

### Model Fitting

The effect of ethanol concentration, time of extraction and temperature on TPC and DPPH radical scavenging activity of the flower of *C. ternatea* was shown in Table 1 whereas all the experimental value of responses were included in the table.

## TPC

The TPC of *C. ternatea* obtained was evaluated by using RSM where all the independent variables were fitted in second-order reduced model equation as shown below.

(1) $$\text{Y}1=40.54-4.81\;{\text{X}}_{1}-0.74\;{\text{X}}_{2}+2.4\;{\text{X}}_{3}+2.4\;{\text{X}}_{1}\;{\text{X}}_{3}-4.19\;{\text{X}}_{12}$$

The result of TPC of *C. ternatea* obtained was evaluated by using ANOVA generated by RSM Design Expert 7.1.5 as the value of *R*^2^ tabulated in Table 2. The model was shown to be significant with its *p*-value < 0.0001 and the *p*-value of lack of fit was 0.0951 (*p* > 0.05) which shown to be non-significant. These conditions indicated that this model can well fit the data and accurately predict the responses. The analyses of variance were performed in determining the significance of independent variables on the responses. In this case X_1_, X_3_, X_1_ X_3_, and X_12_ were significant model terms with *p* < 0.05. Thus, other non-significant model terms which were X_1_X_2_, X_2_ X_3_, X_22_ and X_32_ not being included in the ANOVA table above as they had been reduced to improve the model.

The *R*^2^ value for this response variable was 0.9114 (Table 2), which was greater than 0.80 and it indicated that the model and data obtained were well-fitted and thus reaction can be best explained by regression model. Meanwhile, the adjusted *R*^2^ value was 0.8744 whereas the predicted *R*^2^ value was 0.6563 which were not close with each other probably due to large block effect although model reduction had been done. The C.V of 6.34%, which was less than 10% indicates that the response’s reliability and precision was very high. To measure the signal of noise in this experiment, adequate precision was used. The ratio of 15.848 showed an adequate signal as it was greater than 4. Therefore, this model can be used to navigate the design space. The study done by Kamkaen and Wilkinson (2009) showed the TPC in *C. ternatea* flower extract was 1.9 mg g^−1^ extract as gallic acid equivalents without optimisation by RSM method.

### DPPH Free Radical-Scavenging Assay

The antioxidant activity of *C. ternatea* was analysed by DPPH free radical-scavenging assay and results obtained were evaluated by using RSM where all the independent variables were fitted in second-order reduced model Equation 2.

(2) $$\text{Y}2=63.87-8.23{\text{X}}_{1}+0.59\;{\text{X}}_{2}-2.53{\text{X}}_{3}-4.36\;{\text{X}}_{12}-2.83{\text{X}}_{32}$$

The model was shown to be significant with its *p*-value < 0.0001 and the *p*-value of lack of fit was 0.0989 (*p* > 0.05). These conditions indicated that this model can well fit the data. X_1_, X_3_, X_12_, and X_32_ were shown to be significant model terms. Thus, other non-significant model terms with *p* > 0.05 which were X_1_ X_2_, X_2_ X_3_, X_1_ X_3_ and X_22_ are not included in the ANOVA table above as they had been reduced to improve the model. However, X_2_ was not a significant factor with its *p*-value of 0.5649 but it remained in the model because this factor was one of the main analysed factors. This condition was as the same as in the TPC discussed above.

The *R*^2^ value for this response variable was 0.8948 (Table 3), which was greater than 0.80 and it indicated that the model and data obtained were well-fitted. Thus, reaction can be explained by regression model. In addition, the value of adjusted *R*^2^ and predicted *R*^2^ were 0.8510 and 0.4757 respectively which were shown to be quite large in difference due to large block effect. The C.V of 6.23% showed that the response’s reliability and precision was very high as the value was less than 10% according to Latip *et al*. (2015). To measure the signal of noise in this experiment, adequate precision was used. The ratio of 12.593 showed an adequate signal as it was greater than 4. Therefore, this model can be used to navigate the design space.

### Analysis of Response Surface

**Figure 1:**
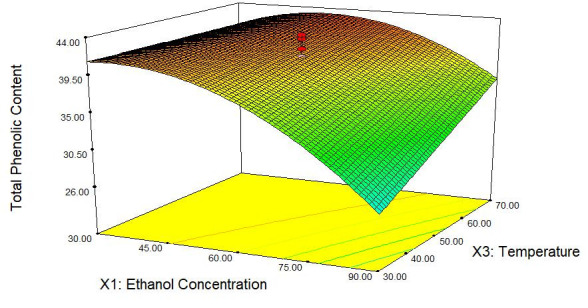
Response surface plot correspond to TPC of *C. ternatea* extract as a function of ethanol concentration and extraction temperature at constant time (90 min).

**Figure 2:**
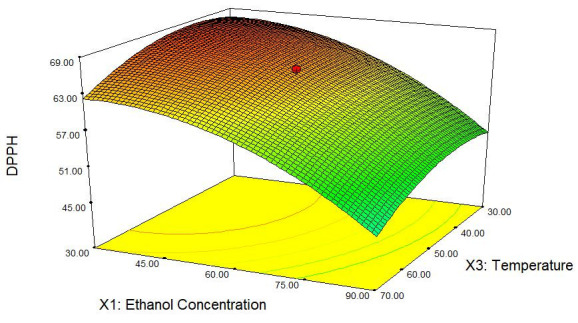
Response surface plot correspond to DPPH radical scavenging activity of *C. ternatea* extract as a function of ethanol concentration and extraction temperature at constant time (90 min).

Three-dimension (3D) response plots for TPC and DPPH-free radical scavenging assay of *C. ternatea* as a function of ethanol concentration and temperature are shown in Figs. 1 and 2. The model had been reduced as there was no significant effect on the changes of time toward the TPC and antioxidant activity, respectively.

Response surface plots for TPC and DPPH scavenging activity of *C. ternatea* flower extract above show that ethanol concentration and temperature affected both responses. Ethanol concentration was shown to be the most significant factor in regression model for both TPC and DPPH scavenging activity. The *p*-value was the smallest among other variables (*p* < 0.0001) for both responses. The equations that had been previously showed indicated that ethanol concentration provide negative quadratic effect on TPC which was expressed as gallic acid equivalent (mg GAE/g of dried sample) and DPPH scavenging activity. These responses were increasing at lower ethanol concentration. These findings showed that *C. ternatea* contains high amount of hydrophilic phenolic compounds, causing it to be dissolved highly in solvent with high water content as in the case of polyphenols extraction in *Salvia officinallis* L whereby its TPC maximised at low fraction of alcohol (Dent *et al*. 2013), indicating that the volume of water in water/organic solvent mixtures contribute to high impact on the extraction of polyphenols compare with the solvent itself. Hence, the antioxidant activity in this case would increase at low ethanol concentration because the phenolic constituents extracted help in promoting the antioxidant capacity in *C. ternatea*.

Based on the visualised plots, temperature was significant for both TPC and DPPH scavenging capacity. However, the trends between the effect of temperature on TPC and DPPH scavenging activity were different between each other. According to both figures, the amount of TPC being extracted mounted up when sample was extracted at higher temperature, whereas the antioxidant activity of *C. ternatea* decreased when the extraction temperature increased. As for the TPC, the expected outcome was as being reported by Liyana-Pathirana and Shahidi (2005) whereby TPC should increase with the increment of extraction temperature due to the ability of heat to cause the cell to be permeable thus, increasing the solubility and diffusion of extracted compound while decreasing the solvent viscosity and promoting its transition through solid substrate mass. However, as for DPPH scavenging activity, higher extraction temperature caused the decrease of antioxidant capacity. This result showed that other bioactive constituents may play vital roles in the antioxidant activity of *C. ternatea* other than the extracted phenolic compounds that might be degraded as temperature increased. Research conducted on the stability of anthocyanin toward heat had shown that accumulation is affected by ambient temperature (Kim *et al*. 2017). Therefore, anthocyanin content in these flowers may contribute to antioxidant activity.

Time was kept constant at the middle level in both responses’ plots because it was not significant according to the analysed ANOVA result (*p* > 0.05). According to the chosen range 60 min to 120 min, no significant changes found on TPC being extracted as well as the antioxidant activity. This finding was probably since the suitable extraction time of *C. ternatea* was below 60 min as in the case of optimising extraction parameters of phenolic compound in *Vitis vinifera*, no significant difference (*p* > 0.05) was observed between 60 min to 180 min of extraction time (Benmeziane *et al*. 2014). This condition can be best explained by Fick’s second law of diffusion which predict that there will be final equilibrium between the solute in plant samples’ matrix as well as in bulk solution or extraction solvent (Chew *et al*. 2011). Therefore, further increase of extraction time would not give any effect on the extraction of phenolic content as well as the antioxidant activity.

### Validation of Predictive Model

Based on the result obtained, an optimum extraction condition was being generated by RSM with Design-Expert 7.1.5 software. Table 4 shows the optimum extraction condition, predicted and experimental values of responses on *C. ternatea* extract.

The optimum extraction conditions provided were as follows: ethanol concentration of 36.92%, extraction time at 90 min and extraction temperature at 44.24°C. Therefore, experiment was conducted to validate the optimum condition by adjusting the optimum paremeters to 37% of ethanol concentration, 90 min of extraction time and and 45°C of extraction temperature considering the operating convenience of equipment.

The predicted values for TPC and DPPH scavenging activity were 41.6 mg GAE/g dried samples and 68.12% scavenging activity, respectively with 0.903 desirability. According to the conducted validation test, the experimental result of TPC was (41.17 ± 0.5) mg GAE/g dried samples and (63.53 ± 0.95)% inhibition for DPPH scavenging activity. Therefore, the percentage difference between the predicted and experimental values of TPC and DPPH scavenging activity were 1.03% and 6.74%, respectively while the *p*-value were 0.25 (*p* > 0.05) and 0.14 (*p* > 0.05), respectively. These findings were good indication that the response model can accurately reflect the expected optimisation in accordance to less than 10% of percentage differences and *p* > 0.05 showing that there was not significance difference between the experimental and predicted values of both TPC and DPPH scavenging activity.

## TFC

Based on the optimum extraction condition obtained, the TFC of *C. ternatea* flower extract was determined by using aluminium chloride method to analyse the relationship between TPC and flavonoid content of the flower’s extract. Result was expressed as mg quercetin/g dried sample with the equation of standard curve of *y* = 0.0002*x* – 0.0467 and *R*^2^ = 0.987.

The amount of TFC of *C. ternatea* obtained from the optimum extraction condition was (187.05 ± 3.18) mg quercetin/g dried sample which was higher than the value of TPC. Chayaratanasin *et al*. (2015) demonstrated that the content of total phenolics was higher than flavonoids in *C. ternatea* flower extract which were 53 ± 0.34 mg gallic acid equivalents/g dried extract and 11.2 ± 0.33 mg catechin equivalents/g dried extract, respectively without optimisation by RSM method. Theoretically, the amount of TPC should be higher than that of total flavonoid content. However, different types of species may have different structure of phenolic compounds and flavonoids. In addition, the TPC measured by different methods might result in output difference (Katsube *et al*. 2004). Therefore, the Folin-Ciocalteu procedure only cannot give the full phenolic constituents extracts. Hence, the reason behind the higher TFC over TPC was since other phenolic contents cannot be quantified by this single FC method. The overall phenolic contents that promote the quantification of flavonoid compound cannot be determined due to lower phenolic content reading than TFC.

However, according to Kaisoon *et al*. (2011), plant extracts with higher flavonoid do not necessarily contain high amount of TPC. The evidence was based on a research conducted by Stanković (2011) whereby the value of TFC of *Marrubium peregrinum* L. extract was higher than its TPC; i.e. (51.33 ± 0.793) mg RU/g extract and (33.51 ± 0.616) mg GAE/g extract, respectively.

### Total Anthocyanin

As anthocyanin is one of the flavonoid compounds in various plants, this analysis was conducted to analyse the presence of anthocyanin in petals of *C. ternatea* due to its purple colour. Result obtained for total anthocyanin in *C. ternatea* was (28.60 ± 0.04) mg L^−1^. Siti Azima *et al*. (2014) found that an amount of total anthocyanin *C. ternatea* flower from Perlis was 23.52 mg g^−1^. The different sampling location of *C. ternatea* flower may reflect the value of total anthocyanin.

Anthocyanins were also considered an antioxidant component in *C. ternatea* (Yang & Zhai 2010). Their stability strongly depends on their structure, pH, light and temperature (Mazza & Miniati 1993). The extractable total anthocyanin from *C. ternatea* extract has proved that the current technology can make indicator from petals of *C. ternatea* due to the ability of anthocyanin to change colour in different pH (Chen & Gu 2013). Moreover, as discussed above, other than phenolic and flavonoid compounds, anthocyanin is an antioxidant constituent in *C. ternatea* that might aid in the reading of DPPH scavenging activity assay in this study.

## CONCLUSION

As a conclusion, RSM had been shown to be able to identify the optimum extraction condition for *C. ternatea* and analyse the significance effect of independent variables chosen on the responses. The time range did not affect the extraction of *C. ternatea* in this study (*p* > 0.05). The time had been kept constant which was at the middle level (90 min). Thus, the optimum condition generated by CCD of RSM was 36.98% of ethanol concentration, 90 min of extraction time and 44.27°C of extraction temperature. However, this optimum condition had been adjusted to 37% of ethanol concentration, 90 min of extraction time and 45°C of extraction temperature in accordance of operating convenience of equipment. An experiment was conducted to validate the optimum condition. The experimental values obtained for both TPC and DPPH scavenging activity clearly agreed with the predicted values. Nevertheless, the slightly lower experimental result of TPC and DPPH scavenging activity might be due to minor adjustment of optimal values of variable generated by RSM. Upon the optimum extraction condition, the extracted total flavonoid and anthocyanin of *C. ternatea* had clearly showed that this flower can also be considered as natural source of phenolics and flavonoid compounds that contribute to its antioxidant activity.

Hence, optimisation is important to be conducted in large-scale industrial activity which involves the enrichment of their products with antioxidant compounds as wide variety of factors influence the extraction yield of plants. Therefore, we should further study other parameters that affect the extraction yield of phenolic content and antioxidant constituents such as pH, different types of solvents, and solvent to sample ratio, to better optimise extraction. The range of independent variables chosen in this experiment should be wide to better analyse the effect of each factor (i.e. ethanol concentration, time and temperature) on the extraction of phenolic content and antioxidant activity.

## Figures and Tables

**Table 1:** Experimental values for the TPC and percent inhibition of DPPH radical scavenging activity of *C. ternatea*.

Run	Independent variables			Responses	
	
	Ethanol concentration (%)	Time (min)	Temperature (°C)	TPC (mg GAE/g dry sample)	DPPH assay (% inhibition)
1	60.00	90.00	50.00	42.56	61.96
2	60.00	90.00	50.00	42.67	61.43
3	30.00	120.00	70.00	38.20	60.36
4	30.00	60.00	30.00	38.07	64.43
5	90.00	60.00	70.00	37.00	50.49
6	90.00	120.00	30.00	26.66	51.61
7	30.00	60.00	70.00	40.13	60.21
8	90.00	120.00	70.00	35.65	50.48
9	60.00	90.00	50.00	41.36	60.55
10	60.00	90.00	50.00	43.09	64.41
11	90.00	60.00	30.00	26.14	50.65
12	30.00	120.00	30.00	39.62	67.15
13	110.45	90.00	50.00	20.00	32.57
14	60.00	90.00	50.00	38.15	62.79
15	60.00	140.45	50.00	37.87	68.85
16	60.00	39.55	50.00	43.20	66.34
17	60.00	90.00	83.64	44.91	49.14
18	60.00	90.00	16.36	37.64	62.41
19	9.55	90.00	50.00	40.86	70.35
20	60.00	90.00	50.00	40.37	64.80

**Table 2:** Reduced model *R*^2^ values for TPC of *C*. *ternatea* flower extracts.

Std. Dev.	2.39	*R*^2^	0.9114
Mean	37.71	Adjusted *R*^2^	0.8744
C.V %	6.34	Predicted *R*^2^	0.6563
PRESS	265.77	Adequate precision	15.848

**Table 3:** Reduced model *R*^2^ values DPPH free-radical scavenging assay of *C. ternatea* flower extracts.

Std. Dev.	3.68	*R*^2^	0.8948
Mean	59.05	Adjusted *R*^2^	0.8510
C.V %	6.23	Predicted *R*^2^	0.4757
PRESS	809.76	Adequate precision	12.593

**Table 4:** Optimum extraction condition, predicted and experimental values of responses on *C. ternatea* extract.

Optimum values	Independent variables			Response	
	
	Ethanol (%)	Time (min)	Temperature (°C)	TPC (mg GAE/g DW)	DPPH free radical scavenging activity (%)
Predicted	36.92	90.00	44.24	41.60	68.12
Experimental	37.00	90.00	45.00	41.17 ± 0.5	63.53 ± 0.95
Percentage difference (%)				1.03	6.74
*p*-values				0.25	0.14
